# Development and application of Single Primer Enrichment Technology (SPET) SNP assay for population genomics analysis and candidate gene discovery in lettuce

**DOI:** 10.3389/fpls.2023.1252777

**Published:** 2023-08-18

**Authors:** Pasquale Tripodi, Massimiliano Beretta, Damien Peltier, Ilias Kalfas, Christos Vasilikiotis, Anthony Laidet, Gael Briand, Charlotte Aichholz, Tizian Zollinger, Rob van Treuren, Davide Scaglione, Sandra Goritschnig

**Affiliations:** ^1^ Council for Agricultural Research and Economics (CREA), Research Centre for Vegetable and Ornamental Crops, Pontecagnano Faiano, SA, Italy; ^2^ ISI Sementi SpA, Fidenza (PR), Italy; ^3^ Limagrain - Vilmorin-Mikado, La Ménitré, France; ^4^ American Farm School, Thessaloniki, Greece; ^5^ Perrotis College, American Farm School, Thessaloniki, Greece; ^6^ Gautier Semences Route d’Avignon 13630, Eyragues, France; ^7^ Sativa Rheinau AG, Rheinau, Switzerland; ^8^ Zollinger Conseilles Sarl, Les Evouettes, Switzerland; ^9^ Centre for Genetic Resources, the Netherlands (CGN), Wageningen University and Research, Wageningen, Netherlands; ^10^ IGA Technology Services Srl, Udine, Italy; ^11^ European Cooperative Programme for Plant Genetic Resources (ECPGR) Secretariat c/o Alliance of Bioversity International and CIAT, Rome, Italy

**Keywords:** lettuce, SPET, high-throughput genotyping, genomic diversity, phenotyping, GWAS, candidate genes

## Abstract

Single primer enrichment technology (SPET) is a novel high-throughput genotyping method based on short-read sequencing of specific genomic regions harboring polymorphisms. SPET provides an efficient and reproducible method for genotyping target loci, overcoming the limits associated with other reduced representation library sequencing methods that are based on a random sampling of genomic loci. The possibility to sequence regions surrounding a target SNP allows the discovery of thousands of closely linked, novel SNPs. In this work, we report the design and application of the first SPET panel in lettuce, consisting of 41,547 probes spanning the whole genome and designed to target both coding (~96%) and intergenic (~4%) regions. A total of 81,531 SNPs were surveyed in 160 lettuce accessions originating from a total of 10 countries in Europe, America, and Asia and representing 10 horticultural types. Model ancestry population structure clearly separated the cultivated accessions (*Lactuca sativa*) from accessions of its presumed wild progenitor (*L. serriola*), revealing a total of six genetic subgroups that reflected a differentiation based on cultivar typology. Phylogenetic relationships and principal component analysis revealed a clustering of butterhead types and a general differentiation between germplasm originating from Western and Eastern Europe. To determine the potentiality of SPET for gene discovery, we performed genome-wide association analysis for main agricultural traits in *L. sativa* using six models (GLM naive, MLM, MLMM, CMLM, FarmCPU, and BLINK) to compare their strength and power for association detection. Robust associations were detected for seed color on chromosome 7 at 50 Mbp. Colocalization of association signals was found for outer leaf color and leaf anthocyanin content on chromosome 9 at 152 Mbp and on chromosome 5 at 86 Mbp. The association for bolting time was detected with the GLM, BLINK, and FarmCPU models on chromosome 7 at 164 Mbp. Associations were detected in chromosomal regions previously reported to harbor candidate genes for these traits, thus confirming the effectiveness of SPET for GWAS. Our findings illustrated the strength of SPET for discovering thousands of variable sites toward the dissection of the genomic diversity of germplasm collections, thus allowing a better characterization of lettuce collections.

## 1 Introduction

Recent years witnessed astonishing advancements in the development of cutting-edge technologies for next-generation sequencing (NGS), opening new frontiers for investigating the genomic diversity of crops (Van Treuren and van Hintum, 2014; Onda and Mochida, 2016). The availability of reference genome sequences and the progress in the field of bioinformatics made it possible to implement high-throughput genotyping methods capable of massively detecting single-nucleotide polymorphisms (SNPs). Being highly abundant across the genome and given their biallelic nature (Wendt and Novroski, 2019), SNPs offer the opportunity to be processed in automated pipelines providing a high resolution in the analysis of population structure and genetic ancestry, enabling furthermore a high-density scan of variants underlying complex traits. Different techniques for the identification of polymorphisms either in specific sites or randomly have therefore been developed. Among these, arrays based on customized oligonucleotide (allele-specific) probes hybridized on solid supports (Tripodi, 2022) offer an efficient technology combining a robust allele calling rate with lower investments in terms of library preparation and downstream bioinformatic analyses. However, arrays are affected by ascertainment bias due to the non-arbitrary sampling of polymorphisms and to the low representativeness of samples used to design the SNP panel leading to the exclusion of rare alleles (You et al., 2018). Furthermore, they are not flexible in terms of upgrades, requiring significant costs to increase the throughput.

The possibility to curtail the complexity of genomes and apply NGS, increasing read depth in determined genomic regions, enabled the development of reduced-representation library based-methods (RRL) (Van Tassell et al., 2008). Among these, genotyping by sequencing (GBS) and restriction site-associated DNA sequencing (RAD-seq) have been the most attractive and affordable options for genome-wide SNP discovery and genotyping (Poland and Rife, 2012; Pante et al., 2015). These methods rely on the use of endonucleases to produce short restriction fragments that, after various steps including adaptor ligation, size selection, and amplification, are sequenced providing the frame for SNP discovery (Deschamps et al., 2012; Kim et al., 2016a; Kim et al., 2016b). Despite the potentialities for developing numerous SNPs in comparison to other genotyping methods (e.g., microsatellites and arrays) and the advantage of a minor ascertainment bias, the main drawback of both GBS and RAD-seq is the uneven distribution of endonuclease cutter sites in the genome (Peterson et al., 2014). The untargeted detection reduces the possibility to identify polymorphisms within functionally relevant chromosomal regions. Indeed, single genes, gene families, promoters and enhancers, gene clusters, and non-coding genes are the genomic fractions that probably contain polymorphisms that are causative of, or tightly associated with, phenotypic variability.

To enable a more targeted approach on functional diversity, NuGEN Inc. (San Carlos, CA, USA) developed single primer enrichment technology (SPET, Patent US9650628B2) (Amorese et al., 2013), a novel customized and cost-effective technology based on Allegro Targeted Genotyping (Lovci et al., 2018). SPET offers the possibility to perform targeted genotyping of known polymorphisms and to discover new random polymorphic loci, thus combining the benefits of both arrays and RRLs (Scaglione et al., 2019). The technology relies on the previous identification of the sites to be sequenced holding the polymorphisms. Based on information gathered from reference genomes or transcriptomes, the target sites are selected, and short DNA probes of ~40 bases long are designed in the adjacent regions. In addition to sequencing of target sites, the probes enable the detection of closely linked novel polymorphisms within the area surrounding the target. Because it uses single primers, the panel design is straightforward, thus enabling a high capability of multiplexing. The tailored design allows SPET to have superior reproducibility and transferability when compared to the other RRL genotyping methods. In plants, SPET has been applied in maize (*Zea mays* L.), black poplar (*Populus nigra* L.) (Scaglione et al., 2019), oil palm (*Elaeis guineensis* Jacq.) (Herrero et al., 2020), cultivated and wild species of tomato and eggplant (*Solanum* spp.) (Barchi et al., 2019), and peach (*Prunus armeniaca* L.) (Baccichet et al., 2022), showing the power of this method for genotyping germplasm collections and crossing populations. Applications included population structure analyses, phylogenetic investigations, high-density linkage map development, and association mapping analysis.

Cultivated lettuce (*Lactuca sativa* L.) is a commercially important crop belonging to the Compositae (Asteraceae), one of the largest angiosperm families comprising over 1,800 genera and 24,000 species (WFO Plant List, 2023). It is considered a main leafy vegetable, widely appreciated by consumers for the content of fibers and the low-calorie intake (Kim et al., 2016). It also represents a good source of vitamin C, iron, folate, and different health-beneficial bioactive compounds (Kim et al., 2016). Its production in 2020 was estimated to be 27.6 million tons on an area of 1.2 million hectares (FAOSTAT, 2023). The genus *Lactuca* comprises approximately 100 species, of which *L. sativa* and its wild progenitor *L. serriola*, both part of the primary gene pool, represent over 90% of the accessions held in genebanks (van Treuren et al., 2012). Cultivated accessions can be classified into diverse horticultural types based on the morphological characteristics of leaves and stems (Simko, 2009). Lettuce germplasm diversity has been explored using different molecular tools including microsatellites (Simko, 2009; Rauscher and Simko, 2013), anonymous and targeted PCR-based markers (van Treuren and van Hintum, 2009), arrays (Stoffel and van Leeuwen, 2012), and RRLs (Seki et al., 2020; Park et al., 2021; Park et al., 2022) to study genetic relationships within and among horticultural types. In the past few years, several genomic resources have been released including the first draft of the lettuce genome (cv. Salinas) (Reyes-Chin-Wo et al., 2017) and the resequencing of 445 accessions including cultivated lettuce and 12 wild *Lactuca* species (Wei et al., 2021), providing a useful source for assessing and exploiting germplasm diversity through novel marker discovery. The possibility to implement both genomic and phenotypic information in genome-wide association studies (GWAS) paves the way to dissect the genetic basis of complex traits. GWAS enable the identification of genomic regions underlying the variation of traits exploiting the ancient recombination events occurring in unrelated individuals (Huang and Han, 2014). The rapid advances of NGS technologies and computational pipelines make GWAS a powerful approach for candidate gene detection in crops. In lettuce, GWAS using different genotyping platforms for SNP discovery investigated agronomic traits (Kwon et al., 2013), resistances (Lu et al., 2014), and quality-related traits (Sthapit Kandel et al., 2020; Park et al., 2021).

In the present work, we describe the development of the first SPET panel in lettuce and its application for analyzing genomic diversity and population structure. A heterogeneous collection of 160 accessions of *L. sativa* and *L. serriola* was used as a proof of concept to validate the SPET assay. We further investigated the potentiality of SPET for candidate gene identification through GWAS in four main lettuce horticultural traits. The obtained results showed the strength of SPET for lettuce genomics.

## 2 Materials and methods

### 2.1 Plant material

**Figure 1:**
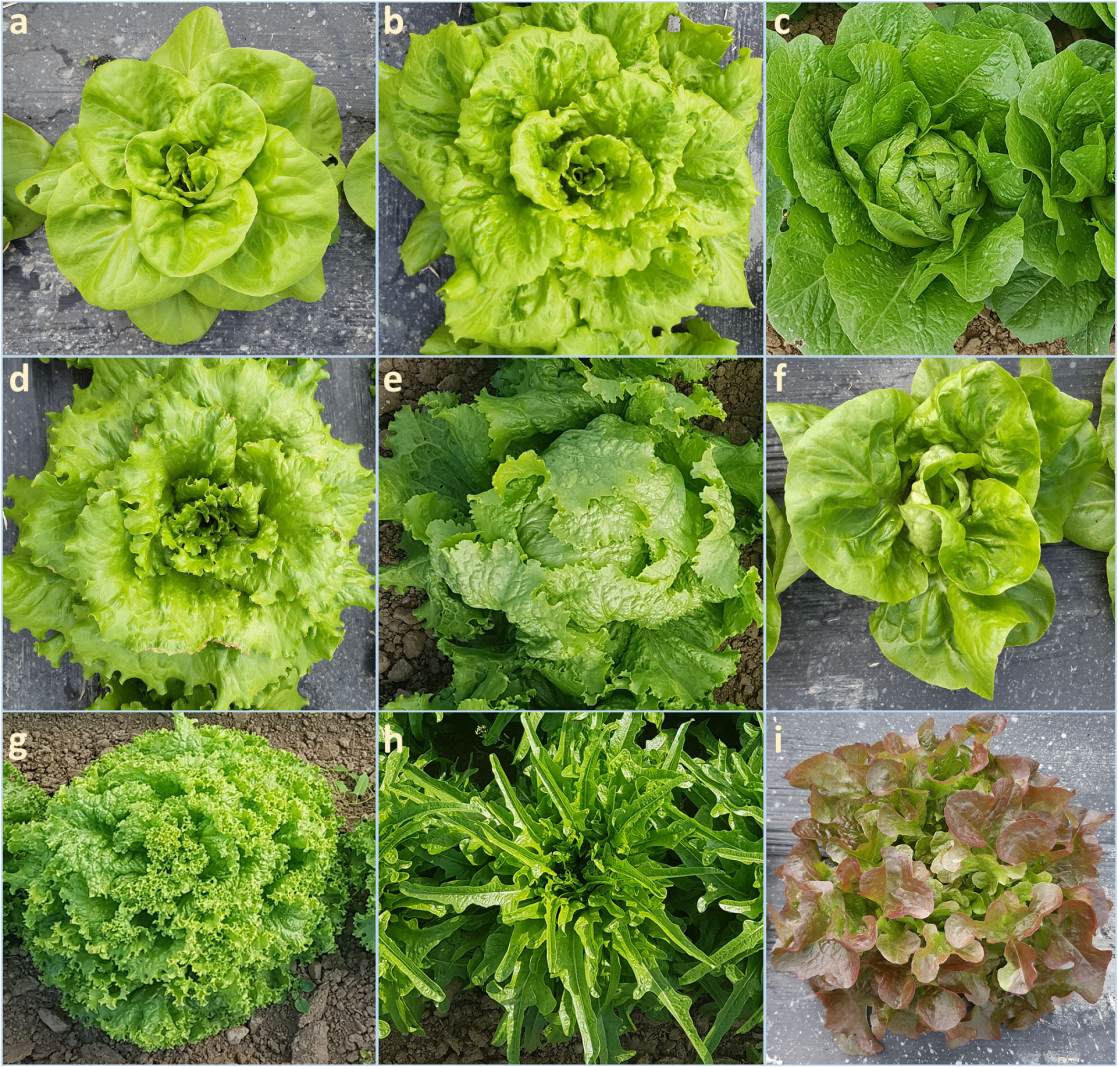
*Lactuca sativa* horticultural types considered in this study. **(A)** EVA_Lsa_00156, Butterhead; **(B)** EVA_Lsa_00166, Batavia; **(C)** EVA_Lsa_00094, Cos; **(D)** EVA_Lsa_00150, Crisp; **(E)** EVA_Lsa_00114, Iceberg; **(F)** EVA_Lsa_00184, Latin; **(G)** EVA_Lsa_00196, Lollo; **(H)** EVA_Lsa_00206, Loose leaf; **(I)** EVA_Lsa_00174, Oak Leaf. Photos provided by Charlotte Aichholz and Tizian Zollinger.

Plant materials consisted of 155 accessions of *L. sativa* and 5 of the closely related wild species *L. serriola*, which were part of the germplasm panel established in the frame of the ECPGR European Evaluation (EVA) Lettuce Network (ECPGR, 2023). Plant materials originated from the germplasm collections of four institutions: the Institute for Plant Genetic Resources “K.Malkov” (Sadovo, Plovdiv district, Bulgaria), the Centre for Genetic Resources, the Netherlands (CGN, Wageningen, Netherlands), the Unité de Génétique et Amélioration des Fruits et Légumes, Plant Biology and Breeding, INRAe (GAFL, Avignon, Montfavet Cedex, France), and the Nordic Genetic Resource Center (Nordgen, Alnarp, Sweden). Genotypes encompassed cultivars, breeding materials, and landraces originating from a total of 10 different countries in Europe, America, and Asia. Different horticultural types were represented (**Figure 1**), including Butterhead (54), Iceberg (46), Cos or Romaine (17), Batavia or Summer/French Crisp (11), Crisp (10), Loose leaf (9), Oak leaf (4), Latin (3), and Lollo (1), as well as wild *L. serriola* (5) (also known as prickly lettuce). A detailed list with all available information on the assayed accessions is provided in **Supplementary Table 1**.

### 2.2 Single primer enrichment technology panel design

For probe design, a dataset including whole-genome resequencing data of 131 *L. sativa* accessions (Wei et al., 2021) was considered (**Supplementary Table 2**). Raw sequence data were retrieved from the FTP site of the China National Gene Bank Sequence Archive (CNSA) repository (Guo et al., 2020). Variants (SNP and INDEL separately) were selected by filtering those present in the dataset with a minimum allele count of 3 (i.e., one homozygous accession and one heterozygous or three heterozygous accessions). The lettuce reference genome (*L. sativa* cv Salinas V8) and its annotation were retrieved from https://lgr.genomecenter.ucdavis.edu/Home.php and all gene coordinates were extended by 5,000 bp upstream and 1,000 bp downstream. All selected genomic variants were intersected with these gene coordinates and labeled as gene-space variants. A panel of 50k target sites was then built by imposing a minimum distance of 3,000 bp for variants on the gene-space and a minimum distance of 200,000 bp in the intergenic regions. After two rounds of design, a final panel of 41,547 targets were successfully identified by unique probes. Each probe consisted of a 40-bp sequence. SNP calling was enabled 460 bp downstream of the probe.

### 2.3 DNA extraction, library preparation, and sequencing

Genomic DNA was isolated from young leaves of a single individual per accession using a NucleoSpin Plant II Mini kit (Macherey-Nagel GmbH & Co. KG., Düren, Germany. DNA concentration was measured using the Qubit 2.0 Fluorometer (Thermo Fisher Scientific, Waltham, MA, USA). Libraries were prepared using the “Allegro Targeted Genotyping” protocol from NuGEN Technologies (San Carlos, CA), using 10 ng/μl of DNA as input and following the manufacturer’s instructions. Libraries were quantified using the Qubit 2.0 Fluorometer, and their size was checked using the High-Sensitivity DNA assay from Bioanalyzer (Agilent technologies, Santa Clara, CA) or the High-Sensitivity DNA assay from Caliper LabChip GX (Caliper Life Sciences, Alameda CA). Libraries were quantified through qPCR using the CFX96 Touch Real-Time PCR Detection System (Bio-Rad Laboratories, Hercules, CA) and sequenced on the Illumina NovaSeq 6000 (Illumina, San Carlos, CA).

### 2.4 Sequence analysis and SNP detection

Demultiplexing of raw sequencing data and base calling (BCL files into FASTQ files) were performed with the Illumina bcl2fastq2 Conversion Software v2.20 (Illumina, San Carlos, CA). Read quality check and adapter trimming were carried out using ERNE v1.4.6 (Del Fabbro et al., 2013) and Cutadapt (Martin, 2011), both with default parameters. Alignment to the reference genome *L. sativa* cv Salinas V8 (Reyes-Chin-Wo et al., 2017) was done using the Burrows–Wheeler Aligner BWA-MEM v0.7.17 (Li and Durbin, 2009) with default parameters and selection of uniquely aligned reads (i.e., reads with a mapping quality >10). SNP calling was obtained using gatk-4.0 (DePristo et al., 2011) following the software best practices for germline short variant discovery. SNP calling was limited to the regions (460 bp) that were previously defined as downstream of each enrichment probe.

All analyses were implemented in GATK Best Practices v4.1.2.0 (Van der Auwera and O'Connor, 2020) and included the following steps: (i) per-sample variants calling on target regions using HaplotypeCaller with default parameters to create a GVCFs file for each sample; (ii) GVCFs consolidation across multiple samples using GenomicsDBImport with default parameters and target intervals in order to improve scalability and speed for further joint genotyping; (iii) joint genotyping using GenotypeGVCFs with default parameters to produce a set of joint-called variants; (iv) Selection of SNPs using SelectVariants and quality filtering of SNPs using VariantFiltration (filter expression used: QD< 2.0 || MQ< 40.0 || MQRankSum< −12.5). A 1,911,467 biallelic SNPs matrix was obtained. The extra filtration of the VCF was performed with bcftools by setting all data points with fewer than five reads in coverage to a missing data genotype (./.) and retaining only records where a minimum of 96 samples reported a coverage above 10 reads. In total, 835,426 SNPs were obtained. For downstream analysis, 81,531 SNP sites were retained with minor allele count = 3, max missing 0.5, minQ = 30, and minor allele frequency = 5%. VCFtools version 0.1.17 (Danecek et al., 2011) was used. Pattern of nucleotide diversity (p) was estimated in non-overlapping sliding windows with a size of 1 kbp in VCFtools. Functional annotation of the identified variants associated genes was performed using SnpEff (version 3.1) (Cingolani, 2022).

### 2.5 Genomic diversity analysis

Genetic diversity summary of the SNP matrix was performed by the Geno Summary tool implemented in Tassel v5.2.15 (Bradbury et al., 2007). Considering the biallelic nature of SNPs, expected heterozygosity according to Hardy–Weinberg equilibrium (*H*) was calculated according to the formula


$$H=1-\rho^{2}-q^{2}$$

where *p* and *q* each represent the frequency of the different alleles for each SNP.

The polymorphic information content (PIC) was calculated according to the formula (Shete et al., 2000)


$$PIC=H-2\times \rho^{2}\times q^{2}$$

Population structure was determined using the model-based ancestry estimation obtained with ADMIXTURE software (Alexander et al., 2015) with K ranging from 1 to 15. One thousand bootstrap replicates were run to estimate parameter standard errors. Tenfold cross-validation (CV) procedure with five iterations was performed, and CV scores were used to determine the best *K* value. Individuals were considered to belong to a specific *K* population if its membership coefficient (qi) was ≥0.5, whereas the genotypes with qi lower than 0.5 at each assigned *K* were considered as admixed. A neighbor-joining phylogenetic tree was built using the Jones–Taylor–Thornton (JTT) model with 1,000 bootstraps. Analyses were conducted in MEGA X software (Kumar et al., 2018). Principal component analysis (PCA) was performed in Tassel v5.2.15 and the biplot was drawn using the ggplot2 R package (Wickham, 2016).

### 2.6 Phenotypic evaluation

The phenotypic traits were surveyed across five locations (Eyragues, Avignon, France; La Ménitré, France; Les Evouettes, Port-Valais, Switzerland; Rheinau, Switzerland; and Thessaloniki, Greece) during the 2020–2022 spring seasons. Plants were grown in a randomized block design with three replicates. Field trials were conducted using the standard agricultural practices for the local area of cultivation. Four traits were assayed including (i) seed color (1 = white/cream, 2 = yellow, 3 = brown, 4 = black), (ii) outer leaf color before bolting stage (1 = yellow green, 2 = green, 3 = gray green, 4 = blue green, 5 = red green), (iii) leaf anthocyanin content before bolting stage (0 = absent, 3 = weak, 5 = medium, 7 = strong), and (iv) bolting time (number of days from sowing to bolting).

### 2.7 Genome-wide association analysis

Genome-wide association analysis was performed in 155 *L. sativa* genotypes. Six models were used including the general linear model (GLM) (Loley et al., 2013), the mixed linear model (MLM) (Zhang et al., 2010), the multi-locus mixed linear model (MLMM) (Segura et al., 2012), the compressed mixed linear model (CMLM) with population parameters previously defined (P3D) (Zhang et al. in 2010), the fixed and random model circulating probability unification model (FarmCPU) (Liu et al., 2016), and the Bayesian-information and Linkage-disequilibrium Iteratively Nested Keyway model (BLINK) (Huang et al., 2019). All models included the population structure as a covariate. The kinship was estimated using the identity by state (IBS) for accounting relationships among individuals. Phenotypic data from independent experiments were implemented. The significance threshold for marker–trait association was determined after Bonferroni multiple test correction with genome-wide α = 0.05. Considering 81,531 SNPs, the marker was considered significant when the *p*-value was less than 6.212 (−log_10_P = 6.133 × 10^−7^). GLM and CMLM were computed in Tassel v 5.2.82 (Bradbury et al., 2007). MLM, MLMM, FarmCPU, and BLINK were calculated with the GAPIT R package (Wang and Zhang, 2021). Manhattan and quantile–quantile (Q–Q) plots for GWAS results were produced using the R package CMplot. The chromosomal location of the genome-wide significantly associated SNPs was displayed using PhenoGram (https://ritchielab.org/software/phenogram). Significant association signals were checked for their physical position on the *L. sativa* (cv. Salinas) V8 genome. The information about predicted genes was downloaded from the Lettuce genome browser v8.0 (https://phytozome-next.jgi.doe.gov/jbrowse/). Underlying genes and their functions were determined according to Reyes-Chin-Wo et al. (2017).

## 3 Results

### 3.1 SPET array

**Figure 2:**
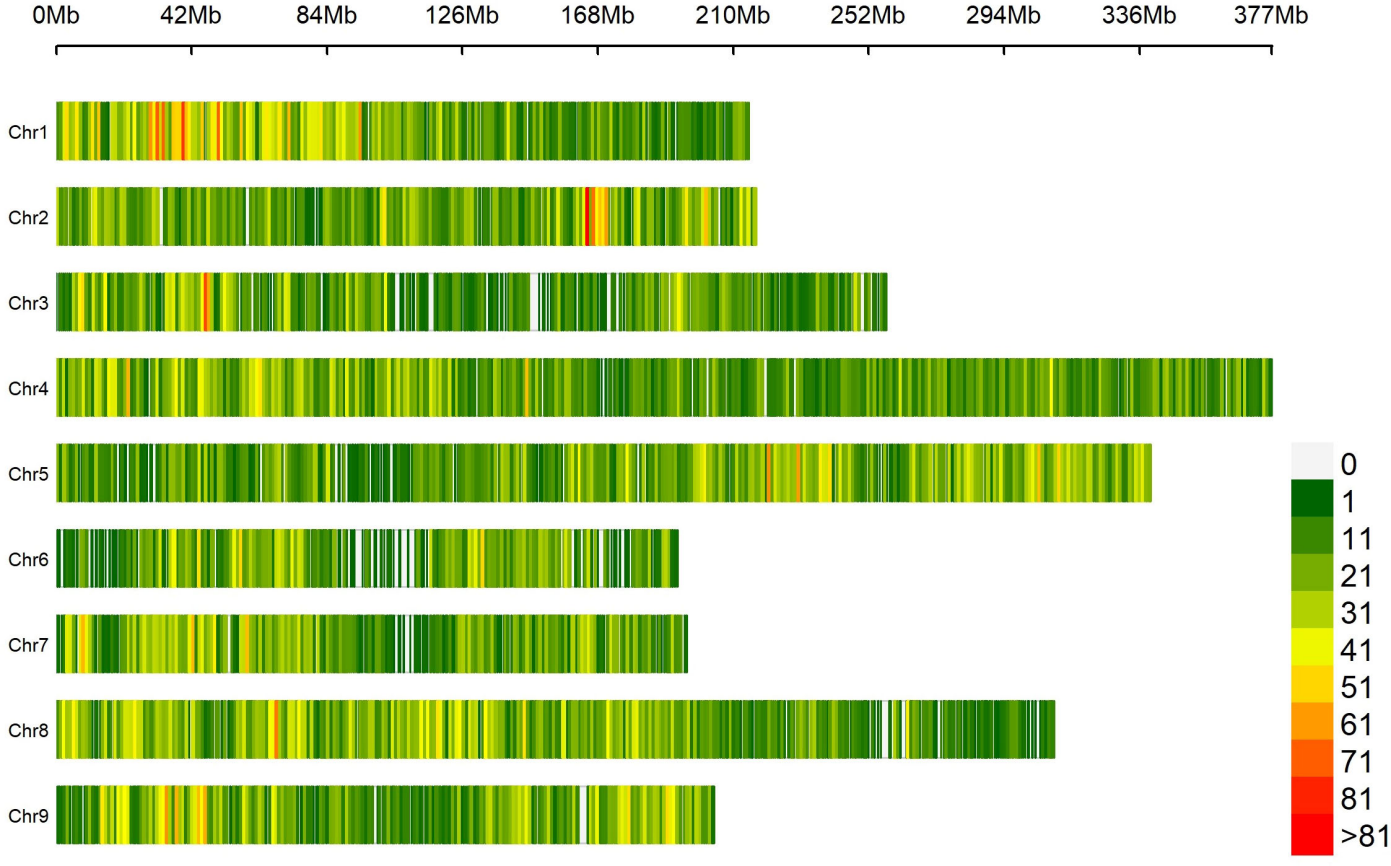
Distribution of 41,547 SPET probes on the nine lettuce chromosomes. The number of SNPs is represented within 1 Mb window size. The horizontal axis shows the chromosome (Chr) length (Mb); each bar represents a chromosome, with Chr 1 at the top and Chr 9 at the bottom. The different colors depict SNP density following the gradient in the legend on the right.

Based on SNP data retrieved from 131 lettuce raw sequences (Wei et al., 2021) and on the alignment to reference genome *L. sativa* cv Salinas V8, 41,547 probes were designed, of which 1,707 (4.1%) were localized in intergenic regions and 39,840 (95.9%) within genes (**Supplementary Table 3**). The average coverage of the total set of probes was 77.1×; for those located within intergenic regions, 93.4×; and for those within genes, 76.6× (**Supplementary Figure 1**). The SPET panel showed an average distribution of one probe per 55.5 kilobase pair (kbp). Regarding inter-probe distance, 27% of the probes were more than 50 kbp apart, while the largest gap was 3.2 mega base pair (Mbp) on chromosome 3 (**Figure 2**). The sequencing of SPET libraries in the 160 study samples produced a total of 668,695,867 paired end raw reads corresponding to an average of 4,179,349 read pairs per sample ranging from 2,000 to 21 million and a mean depth of 79.7× (**Supplementary Table 4**). The mapping rate on the whole genome was on average 88%, and only eight samples had an average below 70% (**Supplementary Figure 2**).

By applying stringent filtering criteria, we identified 81,531 SNPs ranging from 5,291 on chromosome 6 to 13,920 on chromosome 4 (**Table 1**). SNPs were predominantly located within transcript regions, covering over 65% of the gene space in all chromosomes. SNP effect analysis showed that the majority of SNPs (88.08%) have a possible modifier effect, while the rest exhibited low (6.96%), moderate (4.82%), and high (0.14%) impacts (**Supplementary Table 5**). Within gene space, SNPs were mostly localized in upstream and downstream gene regions (27.45% and 16.27%, respectively). SNPs in exons and introns were 11.37% and 7.47%, respectively (**Supplementary Figure 3**). The average density corresponded to one SNP every 28.99 kbp across the nine chromosomes, ranging from 21.48 kbp on chromosome 1 to 36.35 kbp on Chr 6. Across the whole set, PIC values ranged from 0.033 to 0.375 (data not shown) with a mean of 0.240. The minimum average PIC value was encountered on Chr 6 (0.226), while the maximum value was found in Chr 2 (0.258). Chr 6 and Chr 2 exhibited the lowest and highest nucleotide diversity with 4.681^e-4^ and 6.459^e-4^, respectively. On average, heterozygosity was 0.292, reaching values above 0.300 only on chromosomes 2 and 5. The observed transitions/transversions ratio was 2.12 (**Supplementary Figure 4A**). In particular, among transition events, C > T and G > A were the most abundant (18.697% and 18.225%, respectively), whereas C > A and A > T abounded within transversion events (4.826% and 4.506%, respectively). The allele content of the SNP matrix was balanced, being on average represented for 70% by the four nucleotide bases in homozygosity state (**Supplementary Figure 4B**).

**Table 1:** SNP number, distribution in intergenic and genic regions, average distance for each chromosome, polymorphic information content (PIC), nucleotide diversity (**π**), and heterozygosity (*H*).

Chromosome	Chromosome length (bp)	Total SNPs	SNP in genic regions	SNP in intergenic regions	% genic SNP	Average SNP interdistance (kb)a	Max SNP interdistance (kb)	Average PIC	Average p		Average *H*
1	214,780,997	9,995	9,651	344	0.97	21.486	2,434.756	0.245	5.834E-04		0.299
2	217,124,359	9,560	9,149	411	0.96	22.714	1,961.823	0.258	6.459E-04		0.317
3	256,900,232	7,295	6,622	673	0.91	35.220	4,241.704	0.230	5.122E-04		0.279
4	377,162,472	13,920	12,904	1,016	0.93	27.092	1,945.504	0.237	5.407E-04		0.286
5	339,292,695	11,255	10,593	662	0.94	30.148	3,567.503	0.246	5.294E-04		0.301
6	192,650,122	5,291	4,934	357	0.93	36.347	4,532.669	0.226	4.681E-04		0.272
7	195,410,018	6,797	6,470	327	0.95	28.753	2,749.737	0.244	5.048E-04		0.297
8	309,580,090	10,614	10,055	559	0.95	29.164	2,714.974	0.237	5.069E-04		0.287
9	203,529,833	6,804	6,476	328	0.95	29.889	2,562.293	0.235	4.697E-04		0.285

### 3.2 Genomic diversity and population structure

**Figure 3:**
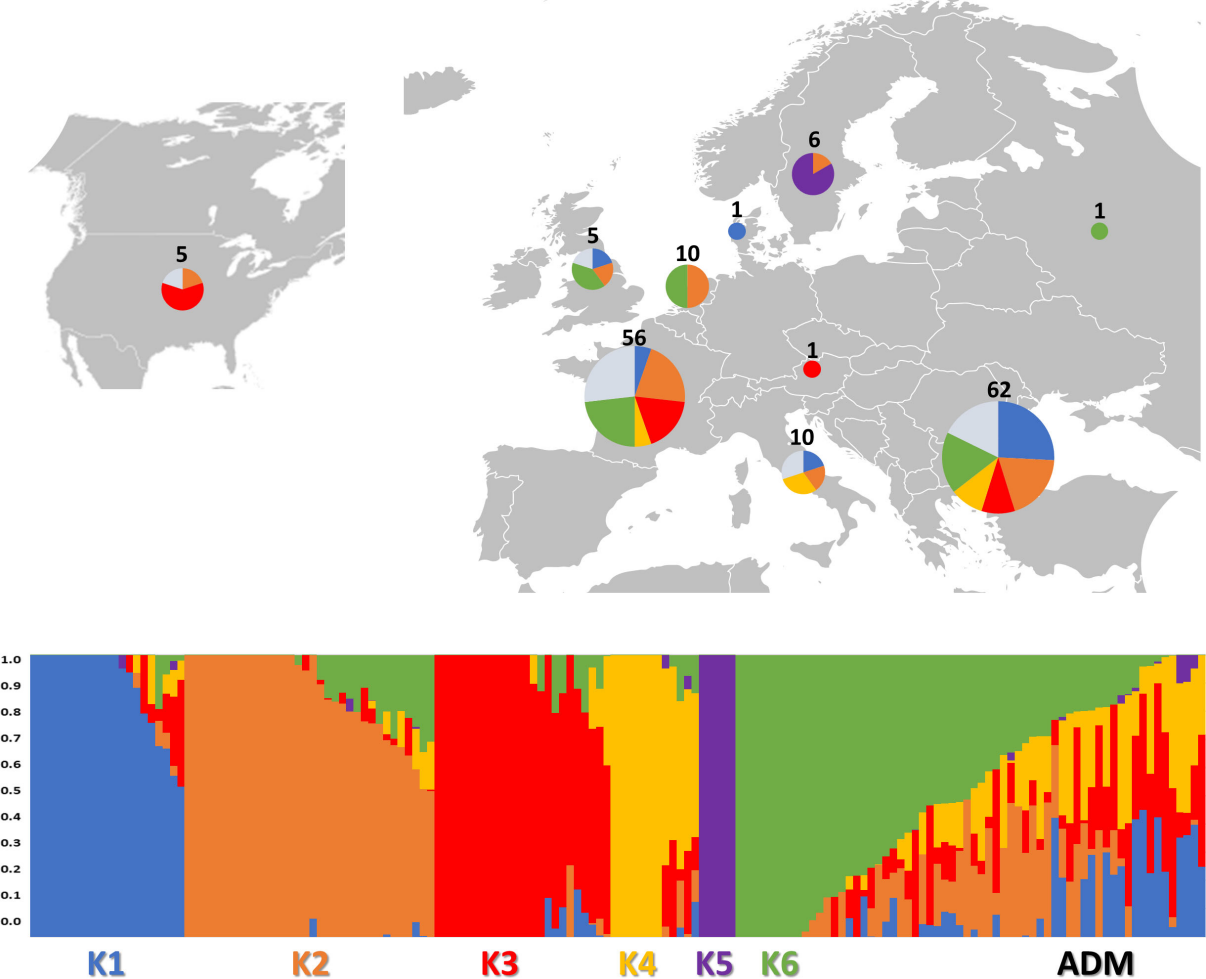
Genetic structure of the 160 study samples using 81,531 SNPs from SPET analysis. On the top: the geographical distribution of the germplasm studied and its subdivision according to the observed K groups; in the pie chart, the proportion of admixed accessions is indicated in ice blue color. On the bottom: bar plot describing the population admixture by the Bayesian approach. Each individual is represented by a thin vertical line, which is partitioned into K-colored segments whose length is proportional to the estimated membership coefficient (*q*). The population was divided into six (*K* = 6) groups according to the most informative *K* value.

An admixture-based clustering model implemented in the software ADMIXTURE (Alexander et al., 2015) was used to infer the genetic structure of the studied germplasm. Using the entire SNP dataset, results of CV error suggested six different clusters (**Supplementary Figure 5**) representing the most likely number of subpopulations (*K*) (**Figure 3**).

The subpopulations reflected to some extent a differentiation based on cultivar typology rather than country of provenance (**Figure 3**; **Supplementary Table 6**). The first cluster (K1) grouped 21 accessions, mostly Iceberg and Cos lettuce types from Bulgaria. Butterhead were mostly grouped in clusters 2 (K2) and 6 (K6) and represented 62% and 67% of the total individuals within each cluster, respectively. The subpopulation 3 (K3) included several Batavia and Crisp types whereas Oak leaf types were included in cluster 4 (K4) together with Iceberg and Loose leaf types. Among the different cultivar types, Iceberg accessions were clustered in several subpopulations. *Lactuca serriola* accessions were grouped separately from the rest in a distinct group (K = 5). Thirty-two accessions belonging to 8 out of the 10 considered cultivar types were classified as admixed, as they showed values for the highest cluster membership coefficient (qi) lower than 0.5. The Fixation Index (*F_ST_*) values, measuring the population (*K*) differentiation based on SNP data, are reported in **Table 2**.

**Table 2:** *F_ST_* values between populations inferred from a model-based ancestry estimation through the ADMIXTURE analysis.

	K1	K2	K3	K4	K5
**K2**	0.409				
**K3**	0.265	0.428			
**K4**	0.313	0.356	0.33		
**K5**	0.676	0.684	0.686	0.592	
**K6**	0.38	0.334	0.397	0.34	0.682

**Figure 4:**
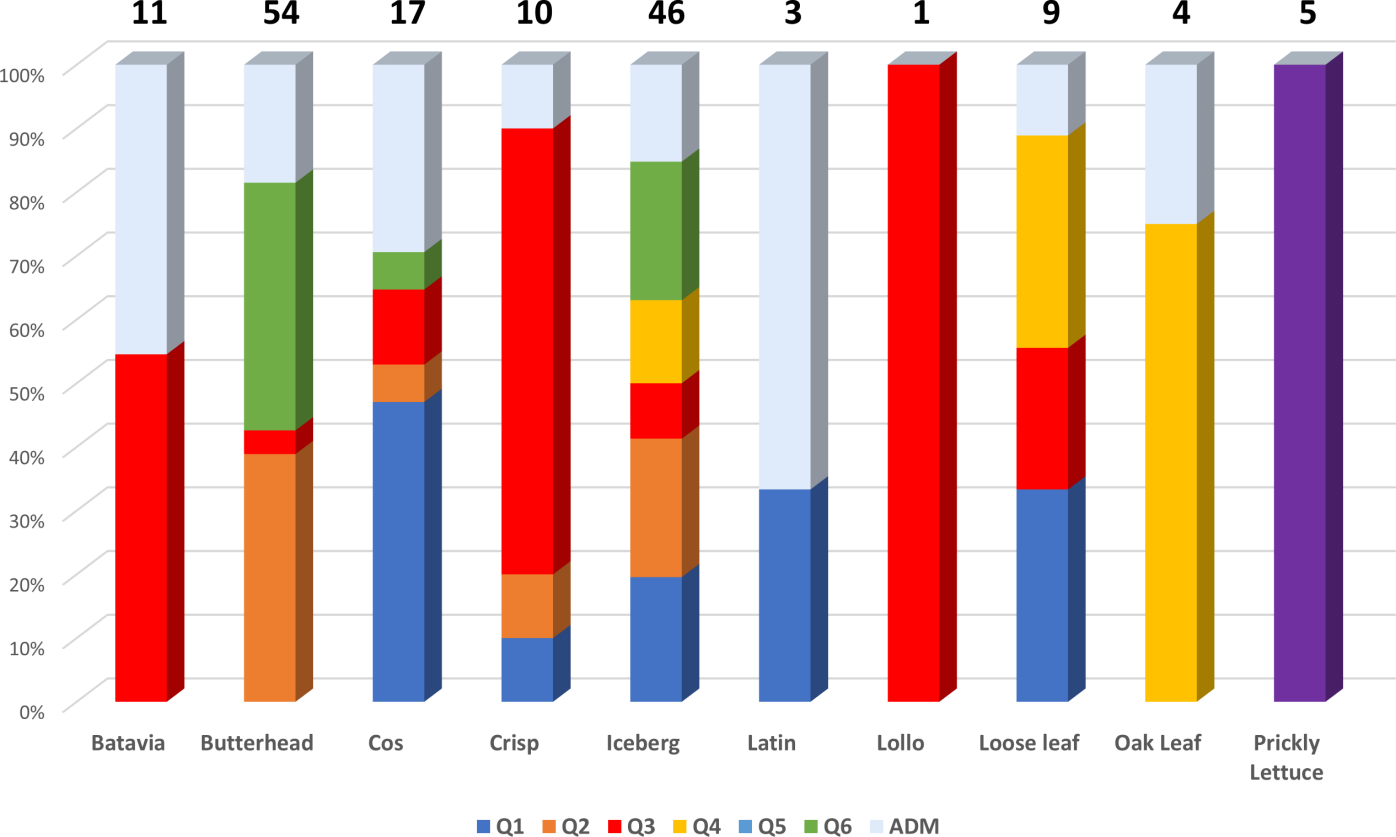
Stacked bar chart of the allele frequency based on Q membership coefficient at *K* = 6. For each cultivar group, the number of accessions is indicated above each bar.

**Figure 5:**
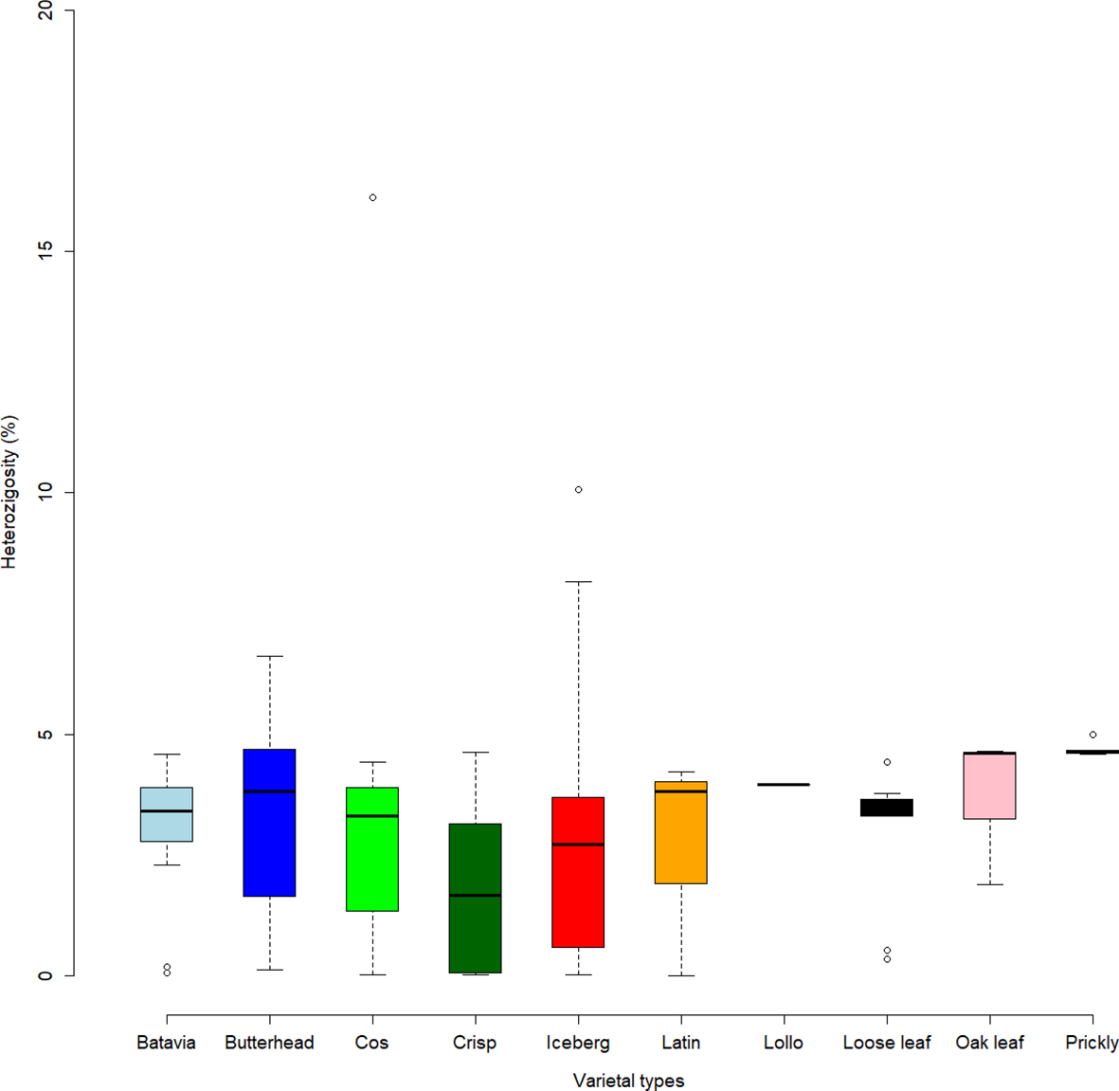
Heterozygosity level (in percentage) of the lettuce accessions. Box plots show median values and quartiles (first and third) of accessions considering the different varietal types.

The highest *F_ST_* values were found between K5 and the other subpopulations, thus confirming the differentiation of the wild *L. serriola* from the cultivated *L. sativa*. The lowest divergence was found between clusters 1 and 3 (*F_ST_* = 0.265) mostly comprising the same type of cultivars. Considering the average *q*-value at *K* = 6 (**Figure 4**), the analysis showed how among the most represented cultivars, iceberg types were included in five out of the six detected clusters while butterheads were included in clusters 2, 3, and 6. Batavia, Crisp, and Lollo as well as Loose and Oak leaf types were mostly represented by clusters 3 and 4, respectively. The average heterozygosity of the accessions was on average lower than 4% in all cultivated variety groups (**Figure 5**). Prickly lettuce accessions showed an average heterozygosity of 4.69% with values ranging from 4.64% to 4.99%. The same trend was observed among different subpopulations based on admixture analysis (data not shown). Twelve accessions belonging to Butterhead (7) and Iceberg (5) exhibited heterozygosity higher than 5% with values up to 6.61% (Butterhead) and 10.08% (Iceberg). Only a single accession, representing the Cos horticultural type, showed a relatively high heterozygosity of 16.11%.

### 3.3 Genetic relationships among accessions

**Figure 6:**
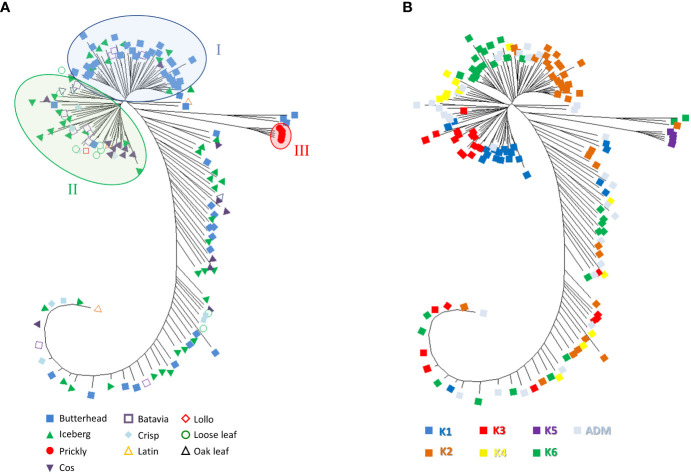
Neighbor-joining phylogenetic tree (radiation style) using 81,531 SNPs from SPET analysis. The evolutionary distances were computed using the Jones–Taylor–Thornton (JTT) model with 1,000 bootstraps. **(A)** Tree with annotated species and horticultural type. **(B)** Tree with annotated grouping revealed by the population structure analysis.

Phylogenetic clustering and PCA were performed to find patterns of genetic variation among accessions. The phylogenetic network using the neighbor-joining method was generally in agreement with Admixture analysis. Two main subpopulations were detected. Group I mostly included butterhead types (**Figure 6A**) from the clusters K2 and K6 (**Figure 6B**).

**Figure 7:**
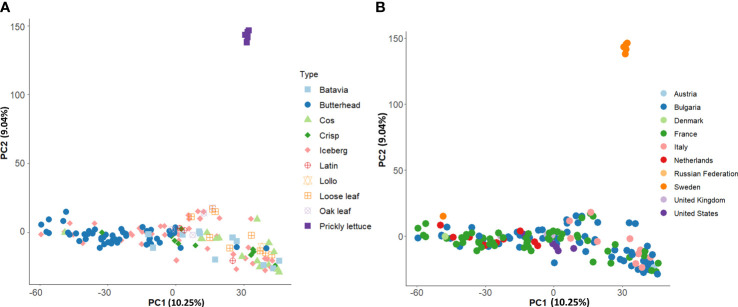
Loading plot in the first two components, showing the genomic diversity of the 160 studied accessions. The PCA was computed with 81,531 SNPs. **(A)** PCA with annotated species and horticultural types. **(B)** PCA with annotated country of origin.

Group II consisted of several icebergs, loose leaf, and cos types from clusters K1, K3, and K4 (**Figures 6A, B**). All prickly lettuce (*L. serriola*) genotypes were grouped closely together according to the cluster K5. The distribution of the accessions in the PCA bi-plot graph corroborated population structure analysis highlighting, among *L. sativa* accessions, a clustering of butterhead types compared to the rest (**Figure 7A**). Prickly lettuce genotypes were grouped apart on the second component, thus confirming the observed subpopulation in the ancestry analysis. A slight differentiation between French and Bulgarian germplasm was observed. Interestingly, several close relationships were found between Italian and Bulgarian accessions. Although more admixtures were found when the geographical provenance was considered, a general differentiation was observed between germplasm retrieved from Western and Eastern Europe (**Figure 7B**).

### 3.4 Genome-wide association analysis

**Figure 8:**
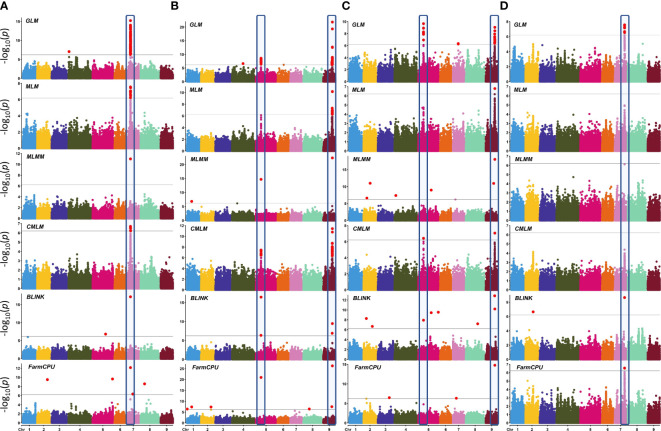
Manhattan plots showing SNP–trait associations (STA) in *L. sativa* using six multi-locus GWAS models. Four horticultural traits are shown: **(A)** seed color, **(B)** leaf anthocyanin content, **(C)** outer leaf color, and **(D)** time of beginning of bolting (bolting time). Analysis has been performed considering 81,531 SNPs on 155 accessions. The black horizontal line indicates a significant threshold (−log10 *p*-value) according to Bonferroni. The *X*-axis indicates the chromosome position. The STA repeatedly identified by three or more GWAS models are highlighted.

**Figure 9:**
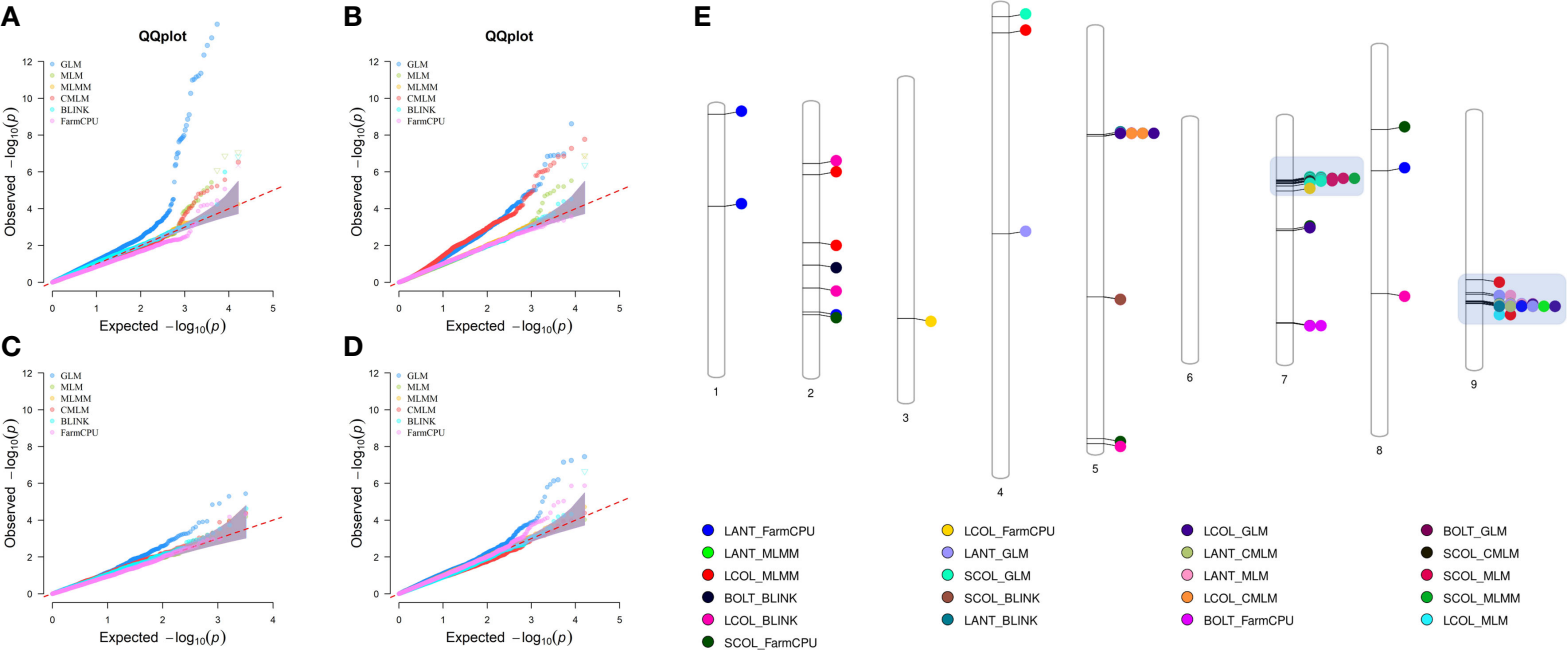
Quantile–quantile plots for six multi-locus GWAS models **(A–D)** and physical position of SNP–trait associations **(E)**. For QQ plots, the order of traits are as follows: **(A)** seed color, **(B)** leaf anthocyanin content, **(C)** outer leaf color, and **(D)** time of beginning of bolting. For chromosomes 7 and 9, the clusters of regions with most STA are highlighted.

Genome-wide association scans using six models detected a total of 306 significant SNP–trait associations (STA) (**Supplementary Table 7**) distributed across all chromosomes except for chromosome 6. The majority of STA were detected for seed color and leaf anthocyanin content: 133 and 117, respectively. Fifty-eight percent of associations were identified with the GLM, whereas among the five multi-locus models used, MLM and CMLM highlighted the highest number of association signals. Only for bolting time was no association found with multivariate models, except FarmCPU. Considering all models, chromosomes 5, 7, and 9 held over 94% of the STA, showing further several colocalizations. Manhattan plots showing the associations, their chromosomal positions, and Bonferroni threshold are shown in **Figure 8**, and Q–Q plots for multi-model GWAS and physical position of STA are shown in **Figure 9**. Two main clusters were found for leaf anthocyanin content and outer leaf color in a 160-kb region at 86 Mbp on chromosome 5 as well as in a 2-Mbp region at 150–152 Mbp on chromosome 9. Furthermore, different significant SNPs were detected on chromosome 7 for seed color in a 3-Mbp region at 49–52 Mbp position and for bolting time at 164 Mbp.

In order to narrow down to potential GWAS hotspots, we considered the top-ranked SNPs within each model (**Table 3**). For seed color, five out of the six models detected the strongest signal at 50.40 Mbp on chromosome 7 in an intergenic region at 19.55 kb to an *Atp-dependent rna helicase DEAH5*. The percentage of phenotypic variation explained (PVE%) by each locus ranged from 0.03% to 45.26%. Only with the CMLM model was the highest peak found 147 kb downstream to the previous one (chromosome 7, 50.54 Mbp) and in correspondence to *Cytokinin dehydrogenase 3*. For leaf anthocyanin content, five models detected a robust association on chromosome 5 at 86.12 Mbp in correspondence to *Phototropin-2* with a PVE% ranging from 4.55% to 15.26%. In addition, all models detected the strongest STA on chromosome 9 at 152.91 Mbp within an *MLO like protein 11*. Also, for outer leaf color, the strongest associations were in both chromosome 5 and 9, at ~27 kbp distance from those identified for leaf anthocyanin content. For leaf color, a *Signal peptidase complex subunit 3B* was the candidate gene identified with GLM, CMLM, and FarmCPU on chromosome 5 at 86.15 Mbp. The three models exhibited a PVE% ranging from 2.78 to 20.32. Furthermore, all models detected the strong STA at 152.88 Mbp on chromosome 9 in correspondence to a *General transcription factor 3C polypeptide 6* , with a PVE% ranging from 10.46% to 47.65%.

**Table 3:** Robust associations detected with a multimodel GWAS for four horticultural traits in a germplasm collection of 155 cultivated lettuce accessions.

Trait	Chromosome	Model*	Position^	Major/Minor allele	MAF^+^	Minor allele effect	PVE^#^	Nearest candidate gene	Candidate gene annotation
Seed color	7	a,b,c,e, f	50,400,650	C/T	0.25	0.48–0.80	0.03–45.26	+19.55 kb	*Atp-dependent rna helicase DEAH5*
	7	d	50,547,653	G/T	0.28	3.33 e-08	0.73	0.0 kb	*Cytokinin dehydrogenase 3*
Leaf anthocyanin content	5	a,c,d,e,f	86,123,750	T/A	0.27	0.30–0.55	4.55–15.26	0.0 kb	*Phototropin-2*
	9	a,b,c,d,e,f	152,909,707	G/A	0.22	−1.12 to −0.5	3.99–23.70	0.0 kb	*MLO like protein 11*
Outer leaf color	5	a,d,f	86,150,826	T/A	0.21	0.10–0.30	2.78–20.32	0.0 kb	*Signal peptidase complex subunit 3B*
	9	a,b,c,d,e,f	152,883,490	A/G	0.26	0.45–0.70	10.46–47.65	0.0 kb	*General transcription factor 3C polypeptide 6*
Bolting time	7	a,e,f	164,434,052	A/G	0.49	−1.67 to −1.47	18.71–48.65	−1.77 kb	*FAR1-related sequence 10*

*a, GLM; b, MLM; c, MLMM; d, CMLM; e, BLINK; f, FarmCPU.

^ Position in base pair (bp) based on the v8 version of the reference genome assembly for L. sativa (cv. Salinas) (Reyes-Chin-Wo et al., 2017).

^+^ MAF, Minor frequency allele (range).

^#^ PVE, Range of percentage variance explained.

For bolting time, only GLM, BLINK, and FarmCPU revealed the strongest STA in an intergenic region at 1.77 kbp from *FAR1-related sequence 10* located on chromosome 7 at 164.43 Mbp. The three models exhibited a PVE% ranging from 18.71% to 48.65%.

## 4 Discussion

### 4.1 SPET development and genomic diversity

In this work, we investigated the effectiveness of SPET as a tool for high-throughput genotyping in lettuce. This method has been developed recently, but so far, very little information about how well it performs in plants is reported. To that end, we developed and validated a novel SNP panel enriched of intraspecific SNPs from 131 resequenced genomes and consisting of over 40,000 probes across the lettuce genome. The potentialities of SPET rely on the high-efficiency enrichment of targeted loci and the high scalability of up to thousands of probes in a single reaction (Scaglione et al., 2019). In addition, it offers the possibility of discovering novel SNPs by sequencing the genomic regions surrounding the target SNPs. Compared to other genotyping strategies for reducing genome complexity, this method offers full control of target sites, thus broadening the investigation of variation within genomic regions with a functional role. Furthermore, the possibility to detect SNPs within probe-defined regions improves reproducibility, thus enabling one to implement and/or compare genomic information from different genotyping experiments. Our main goal was to determine the applicability of SPET for assessing the diversity of a heterogeneous germplasm collection of lettuce including genotypes belonging to different horticultural types with diverse geographic origins. This work was done as part of the ECPGR European Evaluation Network (EVA) with the goal of improving the knowledge of crop genetic diversity and exploiting it to breed more resilient crops that can meet the major problems facing agriculture in the upcoming years (FAO, 2021; ECPGR, 2023). A more efficient use of crop diversity is essential for genetic improvement, management, and conservation of germplasm resources. The sequenced dataset comprised an average of 4 million SNPs per sample, which has been indicated to be adequate for processing several thousands of probes (Scaglione et al., 2019). Compared to the 25K SPET panel reported in peach (Baccichet et al., 2022) and the 5K SPET panel described for tomato, eggplant, and oil palm (Barchi et al., 2019; Herrero et al., 2020), the 40K SPET assay designed in lettuce provides a higher number of SNPs covering up to 96% of gene-rich regions. Our findings demonstrated how effective SPET is compared to other genotyping techniques for detecting SNPs within coding regions. In fact, prior studies in lettuce utilizing genotyping by sequencing revealed that the proportion of SNP loci within genic regions ranged from 0.94% to 27.6% (Park et al., 2021; Park et al., 2022).

We detected over 80,000 high-quality polymorphisms that were analyzed to determine population ancestry, phylogenetic relationships, and principal components among the EVA lettuce accessions. The three approaches were complementary, thus supporting the interpretation of results. In agreement with earlier findings (Park et al., 2021; Park et al., 2022; Simko, 2009; Stoffel and van Leeuwen, 2012), no admixture was found between *L. sativa* and *L. serriola*. Indeed, the accessions of the two species were clearly separated. This evidence promotes the potentiality of SPET for phylogenetic studies, as already observed in aubergine and tomato (Barchi et al., 2019). Population structure and phylogenetic analysis revealed the presence of five distinct subpopulations within *L. sativa* with a variable degree of mixture across cultivar groups, confirming previous studies using both short-read genotyping-based techniques (Park et al., 2021; Park et al., 2022; Stoffel and van Leeuwen, 2012) and microsatellites (Rauscher and Simko, 2013). This could be related to the fact that in lettuce breeding, different horticultural types may be used in the pedigree scheme. In the collection assayed, we found a major clustering of butterhead genotypes when compared to the rest. This tendency contrasted with Park and colleagues (2021), who reported instead a greater separation of iceberg accessions from the other types in a collection of 441 individuals. Despite finding a slight differentiation according to geographical provenance, the effect due to the composition of the diversity panel assayed in terms of horticultural types and represented countries must be considered. Furthermore, for breeding and research materials, the reported origin often matches the places where the selection is carried out, thus providing an additional confounding effect. Several factors could affect the subpopulations enclosed in germplasm collections, such as the management practices occurring in the holding genebanks (e.g., level of heterozygosity retained and duplications), the areas of sampling of materials, or the biological status of accessions. Iceberg types investigated by Park et al. (2021) were mostly patented lines from the USDA, whereas we assayed mostly breeding materials, thus suggesting the presence of accessions still under development. The possibility to discover *de novo* polymorphisms free from any sequencing ascertainment bias and at affordable costs commensurable to other next-generation genotyping methodologies designates SPET as an efficient tool for population genomic analysis in lettuce.

### 4.2 Genome-wide association analysis

The advances in genomics and cutting-edge genotyping technology have contributed to the growing availability of large-scale genotypic data of germplasm resources for various crops. The analysis of the genetic underpinnings of complex traits used in GWAS has benefited greatly from the ability to link phenotypic data to genomic sequence data. GWAS has proven to be an effective method for finding genetic variations that are significantly more common for a specific phenotype in unrelated individuals (Xiao et al., 2022). Owing to the greater number of recombination events occurring in natural populations, the advantage over bi-parental mapping populations depends on a larger genetic base to exploit and on higher map resolution (Han et al., 2020). Over the past years, the GWAS computing efficiency has been improved by developing different multivariate models that consider the family kinship inference and population structure covariates to enhance the power of associations and decrease the rate of false positives (Wang and Zhang, 2021). GWAS has been performed with the aim of investigating the potentiality of the SPET panel for candidate gene detection. To that end, we focused on four main agronomic traits driving the selection of cultivated lettuce cultivars and underlying market and consumer preferences. To test the most likely candidate regions underpinning the variation of the considered traits, different models were implemented. As expected, the GLM detected the highest number of STA in all traits, although this model accumulates several false positives, which are eliminated by incorporating additional correcting factors involving a multi-dimensional genome scan able to simultaneously estimate all marker effects (Wang et al., 2014: Chaurasia et al., 2021). By combining multivariate models, we identified seven candidate regions across chromosomes 5, 7, and 9 for the assayed traits. For seed color, the STA found on chromosome 7 confirmed a previous investigation reporting three associations in a 12-Mbp region spanning 69.87 Mbp to 80.63 Mbp (Kwon et al., 2013). We better refined the position at 50.40 Mbp near *deah5* , an ATP-dependent RNA helicase involved in abscisic acid and stress responses in the acquisition of embryogenic competence (Almeida et al., 2020). The *cytokinin dehydrogenase 3* detected within the association may regulate cell division as well as a large number of developmental events in plants (Schmülling et al., 2003). The two candidates may therefore play a role in seed coat development and color.

The position of STA located on chromosomes 5 and 9 for leaf color traits agreed with previous studies (Zhang et al., 2017; Su et al., 2020; Wei et al., 2021). On chromosome 5, Zhang et al. (2017) reported the lead SNPs for leaf color at less than 150 bp (86,123,627, 86,123,633, and 86,123,651) from the top association for leaf anthocyanin color. In the same study, the association on chromosome 9 was in the same region at 17.45 kb (152,892,248) from the top- ranked STA found in this study. These regions are reported to harbor two genes *RLL2* (Red Lettuce Leaves 2) and *ANS* (Anthocyanin Synthase) that encode key enzymes for anthocyanin biosynthesis. We found four main candidate genes. *Phototropin 2* and *mlo like protein 11* both play a key role in leaf development and physiology. *Phototropin 2* is primarily involved in the reception of light direction in the blade and has been demonstrated to promote leaf expansion and flattening (Legris et al., 2021). In *Pistacia chinensis*, *Phototropin 2* has been reported to be involved in the signal transduction for anthocyanin accumulation during leaf coloration in autumn (Song et al., 2021), whereas in octaploid strawberry, it was involved in anthocyanin accumulation in strawberry fruits (Kadomura-Ishikawa et al., 2013).

The *mlo like protein 11* is part of the large family of proteins that regulates pathogen defense and leaf cell death (Pozharskiy et al., 2022). No previous report indicates any function of *mlo like protein 11* in leaf color. A general transcription factor (*3C polypeptide 6*) was found to be involved in outer leaf color on chromosome 9. In plants, transcription factors regulate secondary metabolism (Vom Endt et al., 2002) and are potential candidates for plant organ pigmentation (Ban et al., 2007; Zhou et al., 2014; Su et al., 2020).

The strongest signals found for bolting time at 164.43 Mb on chromosome 7 confirmed previous evidence. Indeed, several studies consistently supported the importance of chromosome 7 for lettuce flowering control (Kwon et al., 2013; Sthapit Kandel et al., 2020; Lee et al., 2021; Rosental et al., 2021). Despite the exact comparisons of the candidate region not always being possible, owing to the different marker system used (Han et al., 2021), our study supports whole-genome resequencing data findings (Wei et al., 2021), which detected a strong association at 164.5 Mbp in correspondence to *phytochrome c* involved in delaying of flowering. With the same effect, the strong STA found in the present study was near *far1* (far-red impaired response 1), a component of the phytochrome A and putatively involved in regulating light control during the developmental stage (Siddiqui et al., 2016; Liu et al., 2020). *far1* directly activates the expression of the evening gene *ELF4* that plays a key role in the circadian flowering clock. In Arabidopsis, it negatively regulates flowering time in synergy with other FRS (FAR-Related Sequence) and FRF (FRS-Related Factor) genes (Ma and Li, 2018). The variation of *far1* expression has also been reported to regulate shoot growth and flowering time in roses.

The creation of a novel SPET assay in lettuce was described in this work, and its potential for genetic diversity and GWAS research was demonstrated by comparing the results with earlier discoveries using different genotyping technologies. Additional research could weigh the benefits and drawbacks of SPET in comparison to whole-genome short and long read sequencing.

## 5 Conclusion

Here, we presented SPET as an efficient method combining the properties of random complexity reduction techniques and arrays, allowing us to choose a set of gene-associated targeted markers for the accurate characterization of lettuce germplasm. The combination of population ancestry and phylogenetic approaches proved to be effective to better understand the genomic structure of lettuce genotypes. It is evident that the observed diversity patterns reflect the varietal composition of the collection and, to a minor extent, the geographical origin, which can be assumed primary factors underlying the diversification. Given the high marker density, the SPET panel has been used as a proof of concept for genome-wide association analysis to identify genomic regions underpinning the variation of main agronomic traits in lettuce. We confirmed previous findings, refined the genomic position of trait loci, and demonstrated the power of SPET for GWAS. These results will be useful for breeding and selection in lettuce. Further applications may include analysis of genetic relationships among species, management of genebank collections, and genetic fingerprinting for plant variety protection as well as GWAS for other additional important traits in lettuce.

## Data availability statement

The original contributions presented in the study are included in the article/**Supplementary Files**. Further inquiries can be directed to the corresponding authors.

## Author contributions

SG and MB conceived and coordinated the project. PT analyzed genomic data and prepared the draft of the manuscript. MB, DP, IK, CV, AL, GB, CA, and TZ performed phenotyping trials. DS, MB, RvT, and SG jointly designed the SPET array. DS and PT performed bioinformatic analysis. All authors contributed to the article and approved the submitted version.
