# Supplementary material for: Development and application of Single Primer Enrichment Technology (SPET) SNP assay for population genomics analysis and candidate gene discovery in lettuce
Source: Front Plant Sci. 2023 Aug 18;14:1252777. doi: 10.3389/fpls.2023.1252777 (PMC10471991; doi:10.3389/fpls.2023.1252777)
Supplement: Supplementary file 1 [file DataSheet_1.pdf]

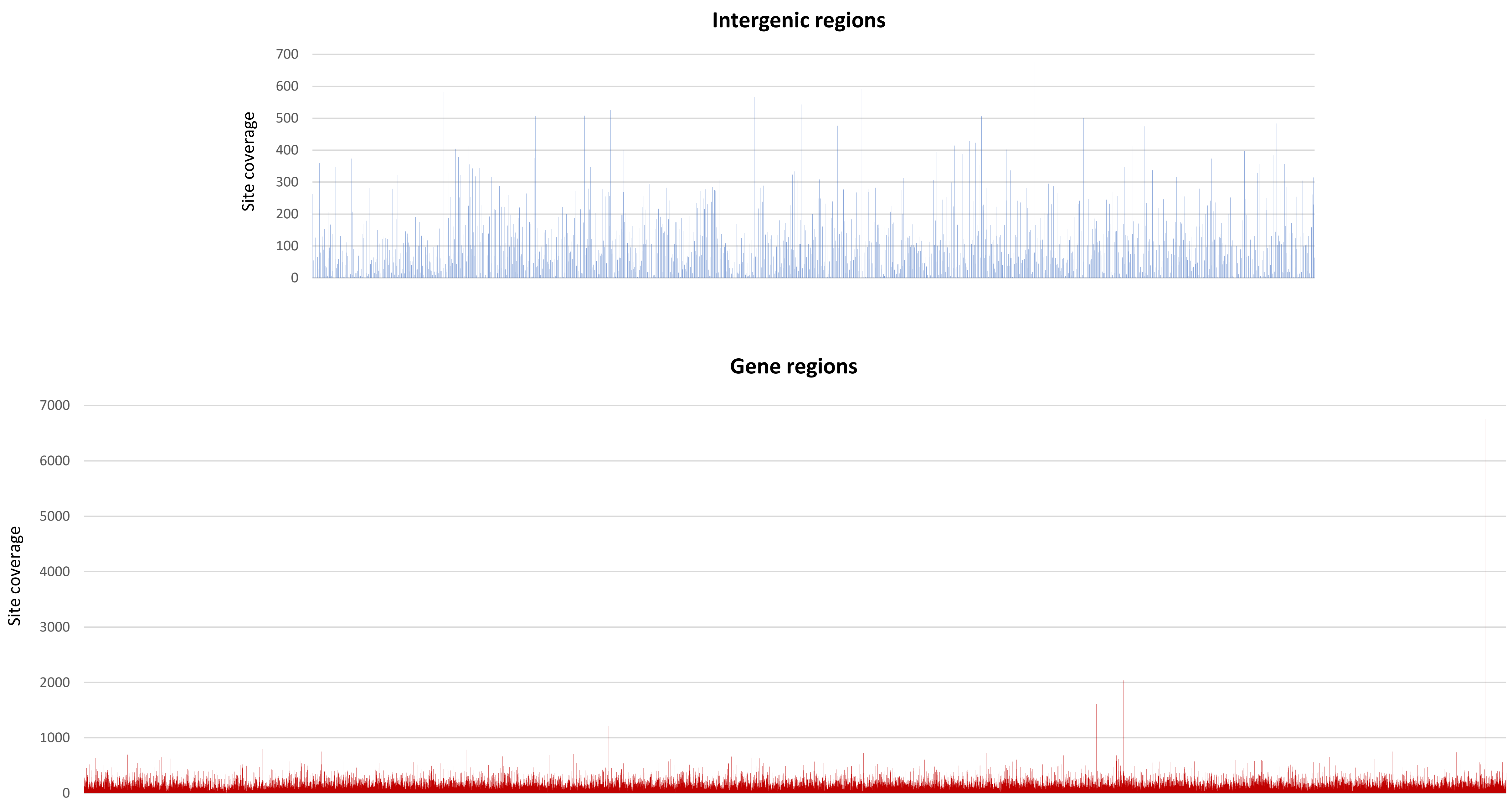

Supplementary Figure 1: Mean coverage obtained in each probe (x-axis) calculated overall samples. For each probe is reported the coverage in intergenic and gene regions

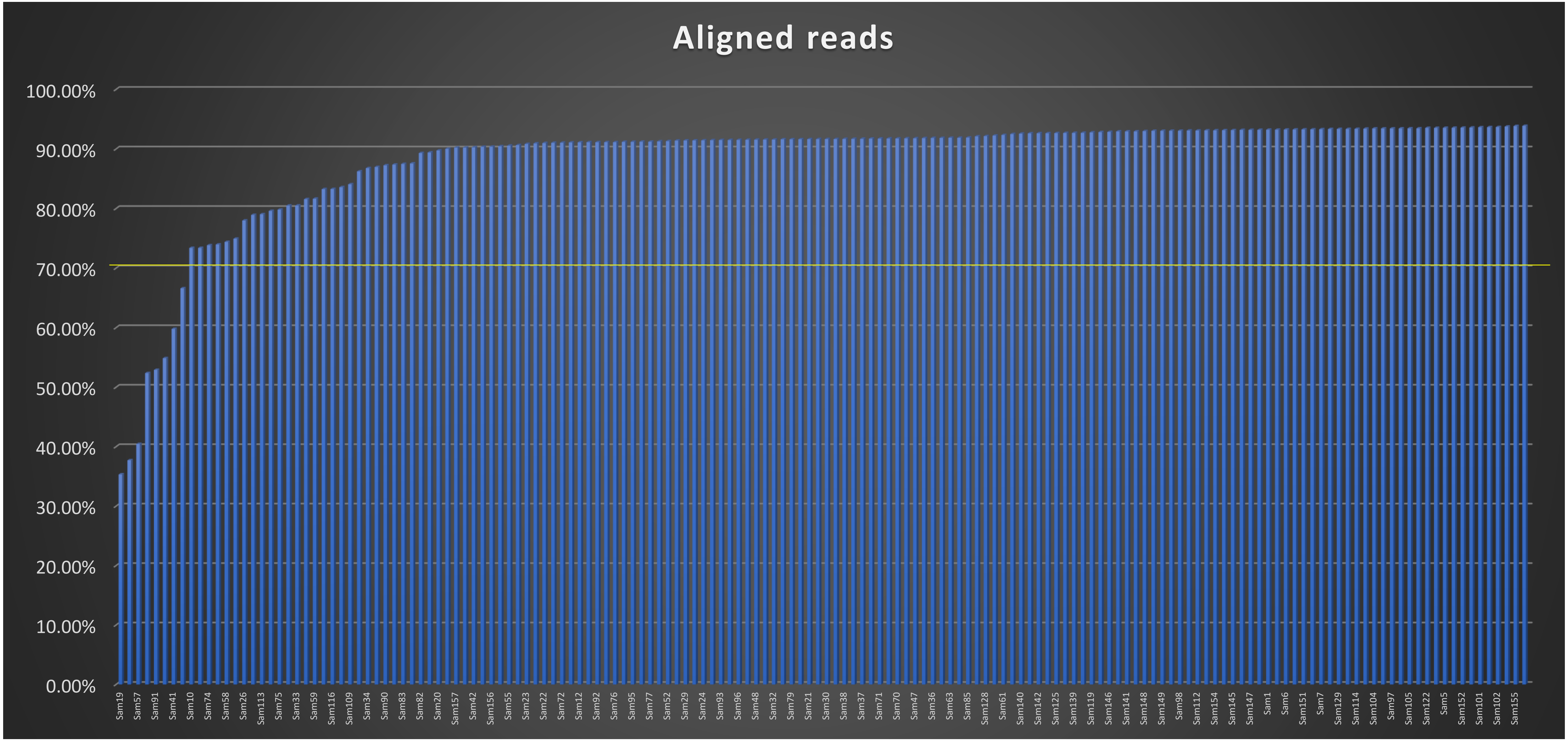

Supplementary Figure 2: Percentage of aligned reads per sample

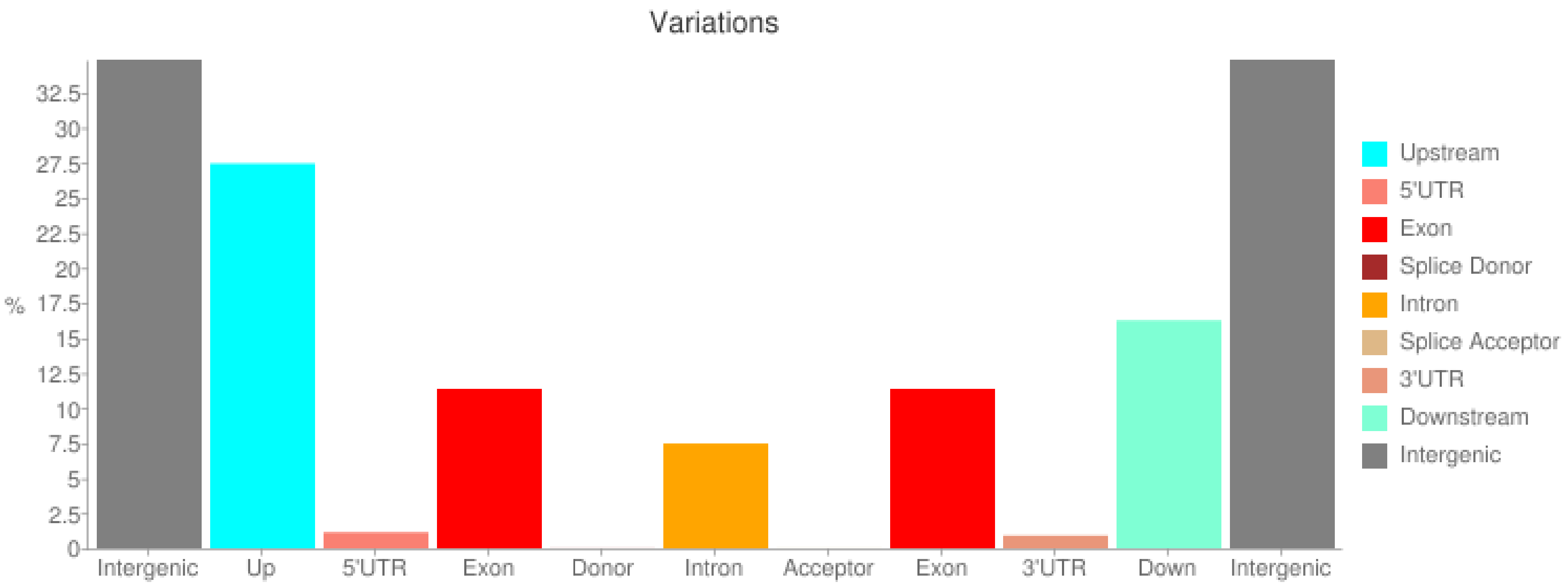

Supplementary Figure 3: Average snpEff output. Shown are average numbers of SNPs affecting specific regions, highlighted in a schematic gene structure shown below the corresponding grouped columns. Most SNPs are up- and downstream of genes (intergenic).

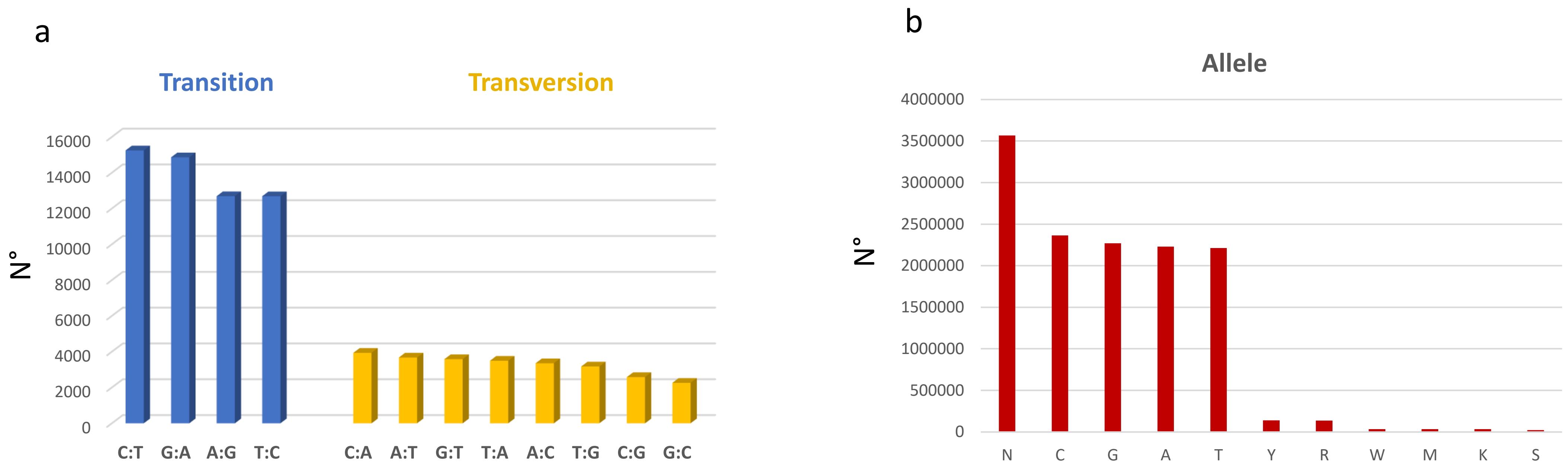

Supplementary Figure 4: a) Bar chart describing the distribution of SNP types divided according to nucleotide substitution as transitions (blue) and transvertions (yellow). b) Type and number of allele in the matrix containing 81,531 SNPs.

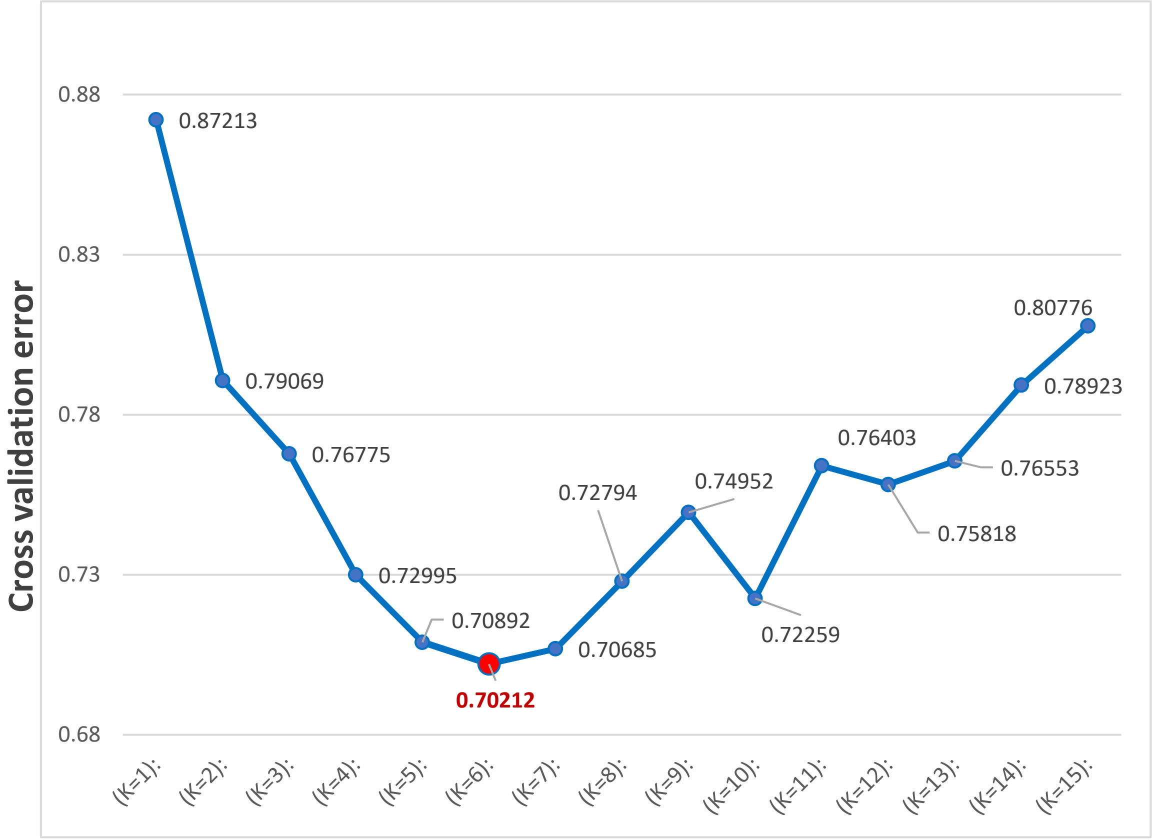

Supplementary Figure 5: Cross Validation error for different admixture models (K=1 to K = 15). The optimal value in red indicates at K = 6, indicates that the 160 lettuce accessions likely form 6 sub-populations.
